# Development of Computational Approach for Analyzing In-Process Thermal-Mechanical Condition during Friction Stir Welding for Prediction of Material Bonding Defect

**DOI:** 10.3390/ma16237473

**Published:** 2023-12-01

**Authors:** Gaoqiang Chen, Huijie Liu, Qingyu Shi

**Affiliations:** 1 State Key Laboratory of Clean and Efficient Turbomachinery Power Equipment, Department of Mechanical Engineering, Tsinghua University, Beijing 100084, China; 2 State Key Laboratory of Advanced Welding and Joining, Harbin Institute of Technology, Harbin 150001, China; liuhj@hit.edu.cn

**Keywords:** friction stir welding, defect prediction, solid-state bonding, thermal-mechanical condition

## Abstract

Unlike the conventional fusion welding process, friction stir welding (FSW) relies on solid-state bonding (SSB) to join metal surfaces. In this study, a straightforward computational methodology is proposed for predicting the material bonding defects during FSW using quantitative evaluation of the in-process thermal-mechanical condition. Several key modeling methods are integrated for predicting the material bonding defects. FSW of AA2024 is taken as an example to demonstrate the performance of the computational analysis. The dynamic sticking (DS) model is shown to be able to predict the geometry of the rotating flow zone near the welding tool. Butting interface tracking (BIT) analysis shows a significant orientation change occurring to the original butting interface, owing to the material flow in FSW, which has a major impact on the bonding pressure at the butting interface. The evolution of the interfacial temperature and the interfacial pressure at the butting interface was obtained to analyze their roles in the formation of material bonding. Four bonding-quality indexes for quantifying the thermal-mechanical condition are tested to show their performance in characterizing the bonding quality during FSW. When the BQI is below a critical value, a bonding defect will be generated. The paper indicates that the simulation-based prediction of a material bonding defect is highly feasible if the developed methodology is extended to quantitatively determine the critical value of the bonding quality index for successful SSB for various alloys.

## 1. Introduction

The modern numerical simulation for welding is advancing to the point that the complex property-concerning features such as microstructure and defects are able to be predicted, which is essential to the process of development for optimizing the weld property. This is also the case for friction stir welding (FSW) [1]. As an advanced solid state welding technology, FSW has unique advantages in joining the high-strength light alloys, such as aluminum alloys, and FSW has been widely used in manufacturing many important structural parts, such as the fuel tanks for aerospace vehicles [2] and the high-speed train body. However, many typical defects in the welds cause unexpected degradation of the weld properties. For example, the material bonding defect, such as root flaw and kissing bond, has been a major problem in friction stir welds, which causes significant degradation of the tensile strength [3,4], fatigue strength [3] and ductility [5] of the welded structure. Generally, much trial-and-error work is required in order to ensure the elimination of the material bonding defects and thus the safety and reliability of the welded structure. Therefore, there is a great need for development of effective computational methodology for analyzing the material bonding induced by the in-process thermal-mechanical condition during FSW, as the time-consuming experimental work for weld defect control can be reduced by computer aided engineering (CAE).

Unlike many other weld cracks, the material bonding defect is a crack array along the unwedded butting surfaces as a result of insufficient solid state bonding (SSB) [6]. The prevention techniques [7,8] and the non-destructive testing [9,10] of material bonding defects in the friction stir welds have been the subject of many experimental studies in the past years. Moreover, due to its size in microns, it is difficult to detect using the current non-destructive testing methods [9]. Although many experimental investigations have been conducted to study the material bonding defect in the welds, the in-process knowledge of the responsible thermal-mechanical condition for material bonding is quite limited. In principle, it is the in-process thermal-mechanical condition that is responsible for successful SSB between the butting surfaces [11]. For the successful SSB of metal surfaces, a certain thermal-mechanical condition is required. Therefore, there are conceptually three steps regarding the computational analysis of the material bonding for defect prediction during FSW, including (1) simulation of the thermal-mechanical condition during FSW, (2) probing the responsible thermal-mechanical state variables for material bonding, and (3) assessment of the thermal-mechanical condition. Here, a brief literature review on the thermal-mechanical simulation of FSW and SSB of metal surfaces by thermal-mechanical processing is provided in order to clarify the computational methodology for the current state of the art.

### 1.1. Thermal-Mechanical Simulation of FSW

The analysis of the thermal-mechanical condition, such temperature, stress and material flow relies on numerical modeling and simulation. Unlike the other modeling approaches [12], computational fluid dynamics (CFD) is widely applied in the thermal-mechanical simulation of FSW, owing to its high computational efficiency and high spatial resolution. Colegrove et al. [13,14] were among the first to develop a 3D CFD-based numerical simulation to quantitatively access the heat transfer and material flow in FSW. In the past decade, the CFD simulation has been extensively strengthened regarding the heat generation model [15,16] and the complex tool profile on the material flow [17,18,19]. It is worth noting that the interfacial friction interaction model is of critical importance in predicting the material flow at the interface between the welding tool and the workpiece. In the literature, the friction interaction model can be divided into two categories, which includes the constant sticking (CS) model and dynamic sticking (DS) model. In most of the previous models, the CS model was employed to implement a fully sticking [16,17] or partial sticking [15,20,21,22] state. The DS model is a relatively new modeling technology, which is undergoing extensive development in order to capture the local contact state at the tool/workpiece interface. Nandan et al. [23] developed a DS model based on the definition of the interfacial velocity, allowing the interfacial friction state to vary as a function of the radial distance. Wang et al. [24] used a contact shoulder radius ratio (CSRR) method to regard the possible change in friction state at the tool/workpiece interface. Colegrove et al. [25] and Liechty et al. [26] demonstrated the use of the shear stress-based model at the tool/workpiece interface for simulation of the in-process material flow during FSW. In previous work, we developed a new DS model via a shear stress boundary condition [19,27,28] based on CFD at the friction interface, enabling automatic transitions between stick and slip states. This new DS model allows analysis of interfacial friction, contact state, heat generation and material flow to be carried out more accurately. The application of new models has improved the predicted thermal-mechanical behaviors, which enables the computational analysis of the responsible in-process thermal-mechanical condition for the material bonding in FSW.

### 1.2. Solid-State Bonding of Metal Surfaces by Thermal-Mechanical Processing

Metal surfaces could establish solid-state bonding (SSB) under certain thermal-mechanical conditions, such as combined thermal-compressing [11]. SSB is responsible for many advanced manufacturing technologies, such as friction welding, porthole die extrusion and cold pressure welding. The published research [29,30,31,32] has focused on experimental studies on the effect of different in-process variables, such as temperature, pressure and plastic deformation, and a theoretical analysis of the SSB process. For successful SSB of metal surfaces, a certain thermal-mechanical condition is generally required [11]. Different from the conventional diffusion bonding, the SSB in FSW occurs in a much shorter time, with relatively high pressure, and is termed rapid SSB. For evaluation of the bonding quality of the rapid SSB, the concept of using a quantitative bonding quality index (BQI) has been widely recognized. Pivnik and Plata [33] proposed a BQI to quantify the responsible thermal-mechanical condition for SSB in porthole die extrusion. This concept of using a BQI has been employed in later studies [34,35,36]. Donati and Tomesani [37] proposed anther BQI for studying the bonding quality in porthole die extrusion, which incorporates the influence of the material flow. In more recent work, Yu et al. [38] proposed a more advanced analytical BQI in their analysis of the bonding quality of extrusion of aluminum alloys, in which the effect of severe plastic deformation on the bonding quality is theoretically considered. In our previous study [39], the collapse of surface asperities in the physical bonding process was described explicitly, and a physics-based analytical BQI is demonstrated in quantifying the SSB quality.

### 1.3. Motivation of the Present Study

Although much investigation has been carried out on development of the computational methodologies for the in-process thermal-mechanical condition in FSW and the quantitative index for assessing the bonding quality, there is still a lack of a computational framework for analyzing the material bonding between the butting surfaces during FSW. In previous studies, Buffa et al. [40] established a criterion of predicting the bonding defect at specific points from a calculated thermo-mechanical field of the FSW process in their finite element analysis. Wang et al. [41] proposed a method to estimate the bonding condition of the FSW joint with calculated strain rate and temperature field using the coupled Eulerian–Lagrangian approach. However, the geometrical evaluation of the original butting interface, where the bonding behavior physically occurs, has not been explicitly considered. Further research is needed to develop a computational procedure and method to quantitatively analyze the material bonding between the butting surfaces in FSW.

This paper presents the development of a simulation-based computational methodology that is capable of quantitatively analyzing the material bonding during FSW by quantitative prediction and evaluation of the in-process thermal-mechanical condition. In particular, CFD simulation is employed to simulate the thermal-mechanical condition during FSW and the bonding quality in the welds is indexed by four different BQIs regarding the in-process thermal-mechanical state variables. The modeling methodologies are described in detail, followed by model verification by experimental measurement. The bonding quality between the butting surfaces is assessed by the BQI, based on the thermal-mechanical variable along the butting surfaces. Finally, the possibility of the prediction of the material bonding defect using the proposed computational analysis is indicated if more supportive data are provided by future studies.

## 2. Computational Approaches

### 2.1. Overview

**Figure 1:**
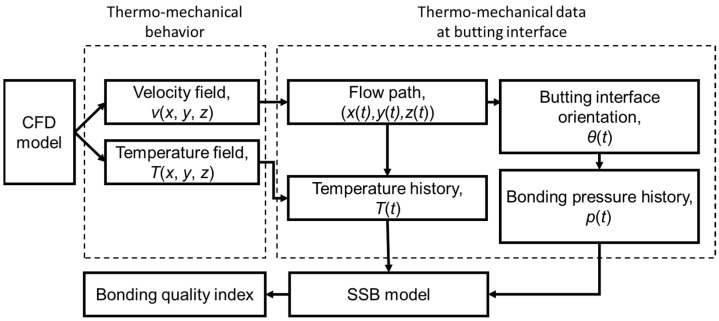
Flow chart of the current study.

In the present study, a CFD model is established to yield the thermo-mechanical behavior of the FSW process. By performing the butting interface tracking (BIT), the thermo-mechanical data at the specific butting interface are probed. Then the data are input into the SSB model to determine the BQI of the welding, as shown in Figure 1.

### 2.2. CFD-Based Thermal-Mechanical Simulation of FSW

In the numerical simulation, the non-Newtonian fluid is adopted to represent the workpiece in FSW, allowing the fully-coupled calculation of transfer of heat, mass and momentum. The general conservation equations of mass, momentum and energy for the non-Newtonian fluid are given by [42],

(1) $$\frac{\partial \rho }{\partial t}+\nabla \cdot \left(\rho \vec{v}\right)=0$$

(2) $$\frac{\partial \rho \vec{v}}{\partial t}+\nabla \cdot \left(\rho \vec{v}\vec{v}\right)=-p+\nabla \cdot \left(\mu (\nabla \vec{v}+\nabla {\vec{v}}^{T})\right)$$

(3) $$\frac{\partial \rho H}{\partial t}+\nabla \cdot (\rho \vec{v}H)=\nabla \cdot (k\nabla T)+S_{V}$$

where ρ is density, μ is viscosity, p is pressure, $\vec{v}$ is velocity and t is flow time, H is enthalpy, T is temperature in K, k is thermal conductivity and $S_{V}$ is a spatial source term regarding the heat flux by plastic deformation. The enthalpy H is defined as $H=\int_{T_{ref}}^{T}C_{P}dT$, where $C_{P}$ is specific heat, and $T_{ref}$ is the reference temperature (27 °C). The governing equations are solved by using a SIMPLE solver by using the commercial CFD software ANSYS Fluent, v15.0 [43]. The user-defined functions (UDF) are used to define many special considerations for FSW.

**Figure 2:**
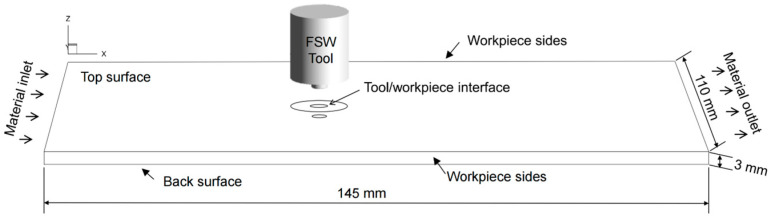
Geometric model in the CFD simulation.

The dimensions of the workpiece are 145 mm × 110 mm × 3 mm (X × Y × Z), which are shown in Figure 2. The geometry of the welding tool is utilized to represent the setup of the welding in the experiment. The workpiece is discretized by using 157,716 hexagonal control volumes. The average size of the grids around the welding tool is taken as 0.2 mm.

The data of density, thermal conductivity and specific heat of the AA2024 are taken as the measured values in a handbook [44]. The fluid viscosity is key to predicting the mass and momentum transfer in the workpiece, especially in the vicinity of the welding tool. The viscosity is taken as dependent on both temperature and strain rate, given by [14],

(4) $$\mu =\frac{\sigma }{3\dot{\varepsilon }}$$

where σ is temperature-and-strain-rate-dependent flow stress and $\dot{\varepsilon }$ is effective strain rate. The flow stress of AA2024 is taken into account by using a nonlinear analytical model, given by [45],

(5) $$\sigma =\sigma_{R}\sinh^{-1}\left[{\left(\frac{\dot{\varepsilon }}{A}\exp\left(\frac{Q}{RT}\right)\right)}^{1/n}\right]$$

where T is temperature, $\dot{\varepsilon }$ is effective strain rate, $A=2.29\times 10^{11}/s$, $\sigma_{R}=47.7\;MPa$ and n=5.46 are the constitutive constants, Q=178 kJ/mol is the thermal activation energy for plastic deformation, and R is the gas constant. These constitutive constants for AA2024 were obtained from Ref. [28]. The flow-stress curve is given in our recent research with an empirical softening regime when the temperature is approaching the solidus temperature. 

The magnitude of the frictional stress at the tool/workpiece interface is given based on Coulomb’s law of friction [28], as

(6) $$\tau_{f}=\mu_{f}{\cdot \sigma }_{n}$$

where the $\mu_{f}$ is the temperature-dependent coefficient of friction (COF) and $\sigma_{n}$ is the interfacial normal stress. The value of the COF is interpolated from the data listed in Table 1.

The first new modeling technology in the present study is the use of a new DS model. The DS model is based on the definition of the interfacial shear stress [27], which allows the automatic transition between a sliding friction state and a static friction (sticking) state. The interfacial shear stress is given by [27]

(7) $$\vec{F}=\tau_{f}\frac{{\vec{v}}_{rel}}{\left\|{\vec{v}}_{rel}\right\|}\tanh\left(\alpha \cdot \left\|{\vec{v}}_{rel}\right\|\right)$$

where $\tau_{f}$ is stress magnitude in Equation (6), ${\vec{v}}_{rel}={\vec{v}}_{tool}-{\vec{v}}_{workpiece}$ is the interfacial relative velocity between the tool and the workpiece, and α is a scaling constant (50 s/m) for implementing a pseudo-static friction state [27]. In the iterative solving process of the conservation equations, the calculated workpiece velocity ${\vec{v}}_{workpiece}$ is updated in each iteration automatically, based on the balance of the friction and the flow stress. 

For comparison purposes, the CS model is also used in the present study. For the CS model, the workpiece was assumed to flow at a specific velocity at the tool/workpiece interface. Similar to References [14,17,22,23], the interfacial workpiece velocity is given by the equation below,

(8) $${\vec{v}}_{workpiece}=\delta \cdot {\vec{v}}_{tool}$$

where δ is a parameter, and ${\vec{v}}_{tool}$ is the tool velocity at the interface. Thus, the interfacial relative velocity is ${\vec{v}}_{rel}=\left(1-\delta \right)\cdot {\vec{v}}_{tool}$. In the present study, δ=1 and δ=0.5 are used, respectively, to represent the fully sticking and sliding friction states.

The localized heat generation around the welding tool is a critical factor in deciding the temperature field. The temperature would determine the flow stress of the workpiece and the friction at the tool/workpiece interface, and thus determine the material flow behavior. Both the friction and the plastic deformation are contributing to the heat generation. Therefore, the heat generation is a fully thermal-mechanical coupled process. Our CFD simulation is able to include this coupling between the thermal-mechanical variables and the heat flux. In the present model, the heat flux is calculated from both friction and plastic deformation. The heat flux by friction is taken as a facial heat flux at the tool/workpiece interface, which is formulated as [27]

(9) $$q_{f}=\eta \cdot \tau_{f}\cdot \left\|{\vec{v}}_{rel}\right\|$$

where $\tau_{f}$ is the magnitude of frictional shear stress in Equation (6) and $\left\|{\vec{v}}_{rel}\right\|$ is the magnitude of the interfacial relative velocity between tool and workpiece, and η=0.7 is the interfacial efficiency representing the fraction of frictional heat into the workpiece [27]. The heat generation from the plastic deformation is considered as a volumetric source term $S_{V}$ in the energy equation in Equation (3). The volumetric heat flux is formulated as [16]

(10) $$q_{p}=\kappa \cdot \sigma \cdot \dot{\varepsilon }$$

where κ=0.6 [27] is the mechanical efficiency representing the fraction of plastic work dissipated as heat, σ is flow stress and $\dot{\varepsilon }$ is effective strain rate.

### 2.3. Butting Interface Tracking for Probing the Responsible Thermal-Mechanical State Variables for Material Bonding

A new modeling technique termed ‘butting interface tracking’ (BIT) is developed in order to assess the responsible thermal-mechanical condition for the SSB between the butting surfaces. In FSW, the butting interface passes the thermal-mechanical processing zone, where high-temperature severe plastic deformation occurs. The simulated 3D thermal-mechanical state variables are denoted as

(11) $$\mathrm{Temperature}:\;T\left(x,y,z\right)$$

(12) $$\mathrm{Velocity}:\;\left\{\begin{array}{c} v_{x}\left(x,y,z\right)\\ v_{y}\left(x,y,z\right)\\ v_{z}\left(x,y,z\right) \end{array}\right.$$

(13) $$\mathrm{Strain}\;\mathrm{rate}:\;\dot{\varepsilon }\left(x,y,z\right)$$

In the present study, 29 points at the Y = 0 plane in front of the welding tool are used to represent the butting interface before welding. For a specific point located at $\left[x_{w0},y_{w0},z_{w0}\right]$ among these points, the flow path is represented by the location of the point ${\left[x_{w}\left(t\right),y_{w}\left(t\right),z_{w}\left(t\right)\right]}^{T}$ as a function of time, which is calculated by integration versus time,

(14) $$\left[\begin{array}{c} x_{w}\left(t\right)\\ y_{w}\left(t\right)\\ z_{w}\left(t\right) \end{array}\right]=\left[\begin{array}{c} x_{w0}\\ y_{w0}\\ z_{w0} \end{array}\right]+\left[\begin{array}{c} \int_{0}^{t}v_{x}\left(x_{w}\left(t\right),y_{w}\left(t\right),z_{w}\left(t\right)\right)dt\\ \int_{0}^{t}v_{y}\left(x_{w}\left(t\right),y_{w}\left(t\right),z_{w}\left(t\right)\right)dt\\ \int_{0}^{t}v_{z}\left(x_{w}\left(t\right),y_{w}\left(t\right),z_{w}\left(t\right)\right)dt \end{array}\right]$$

where the first term ${\left[x_{w0},y_{w0},z_{w0}\right]}^{T}$ is the coordinate of the original point and the second term is the integral term calculating the locations along the flow path. By analyzing all 29 at the original butting interface, the geometric evolution of the butting interface during the welding process is constructed. The 3D orientation ${\left[\theta_{x}\left(t\right),\theta_{y}\left(t\right),\theta_{z}\left(t\right)\right]}^{T}$ of the deformed butting interface can be determined. The in-process thermal-mechanical state variables at the butting interface, such as temperature, strain rate and material velocity, are calculated by interpolation, by using the simulated 3D distributed state variable. The bonding pressure at the butting interface is calculated by

(15) $$p_{bonding}=\left\{\begin{array}{c} p_{welding}\cdot cos\theta_{z}\;\;\;\;,\;\;\mathrm{below}\;\mathrm{shoulder}\\ \;\;\;\;\;\;\;0\;\;\;\;\;\;\;,\;\;\;\;others\;\;\;\;\; \end{array}\right.$$

where $\theta_{z}$ is the angle between the z axis and the normal of the deforming butting interface. $p_{welding}$ is the welding pressure calculated by $p_{welding}=\frac{F_{p}}{\pi R_{sh}^{2}}$, $F_{p}$ is the downward force during welding and $R_{sh}$ is the shoulder radius.

### 2.4. Assessment of the Responsible Thermal-Mechanical Condition for Material Bonding

**Figure 3:**
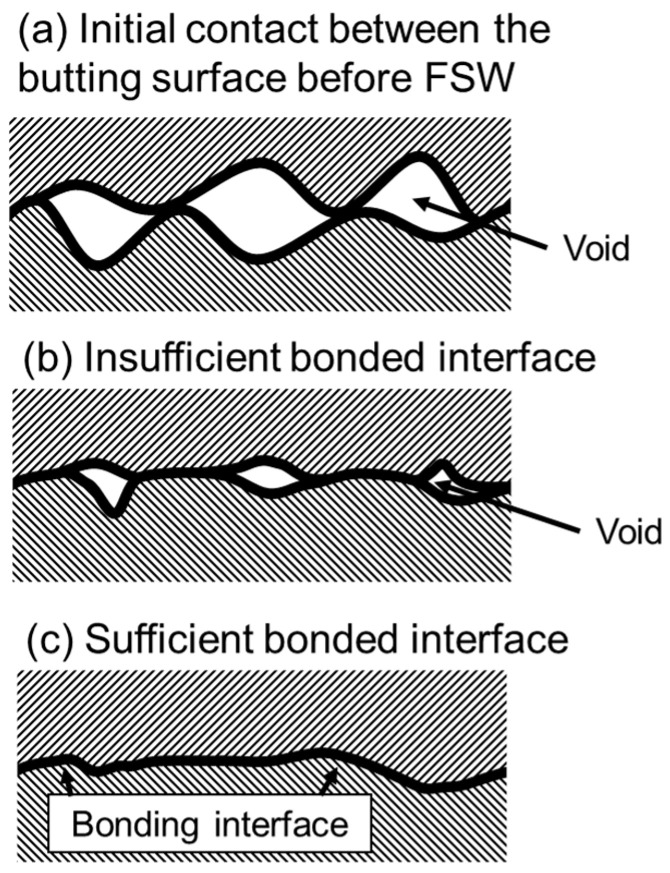
Illustration for the solid-state bonding of metal surfaces.

Following the concepts in the literature [46,47,48], SSB can be described as an interfacial thermal-mechanical processes. Figure 3 illustrates the different bonding states between the butting surfaces. As illustrated in Figure 3a, a very small fraction of the contacting butting surfaces is in real contact, because of the surface roughness. During FSW, the butting surfaces experience high temperature, high pressure and severe plastic deformation; as a result, the surface asperities collapse and thus the butting surfaces will be in intimate contact. As the metallic bond is formed between the positive ions and free electrons and is non-directional and unsaturated, solid-state bonds form between butting surfaces when the distance between the surfaces is below a critical value [38]. If the butting surfaces fail to form a bond, a residual void would exist at the interface, as shown in Figure 3b, which degrades the joint properties. What we expect is a sufficient bonding, shown in Figure 3c, where the interfacial void is eliminated. Therefore, a certain thermal-mechanical condition is required for the closure of the interfacial void and thus the successful bonding between the butting surfaces. 

Four BQIs for quantitatively assessing the thermal-mechanical condition for material bonding quality are used. The four BQIs are all used in the present study to analyze the thermal-mechanical condition along the butting interface. The first BQI is the Q-index proposed by Pivnik and Plata [33], formulated by

(16) $$I_{Q}=\int_{0}^{t}\frac{p_{bonding}}{\sigma }dt$$

where $p_{bonding}$ is the bonding pressure at the butting interface, and σ is flow stress. The term $\frac{p_{bonding}}{\sigma }$ is a normalized pressure, which is generally used in most of the BQIs. The second is the K-index proposed by Donati and Tomesani [37],

(17) $$I_{K}=\int_{0}^{t}\frac{p_{bonding}}{\sigma }v\;dt$$

where v is the flow velocity. The third one is termed tJ-index by Yu et al. [38], given by

(18) $$I_{J}=\int_{0}^{t}k_{0}\frac{p_{bonding}}{\sigma }\dot{\varepsilon }he\;\exp\left(-\frac{Q}{RT}\right)\;dt$$

where $k_{0}=1.0$ is a constant, $\dot{\varepsilon }$ is the strain rate, Q is the activation energy for plastic deformation/diffusion, T is temperature and R is the ideal gas constant. The fourth BQI is termed the F-index, proposed based on Chen et al.’s study [39]. The F-index is formulated as

(19) $$I_{F}=\int_{0}^{t}I_{F}A{\left[\sinh\left(\frac{\alpha p_{bonding}}{I_{F}\sigma_{R}}\right)\right]}^{n}\exp\left(-\frac{Q}{RT}\right)\;dt$$

where α=1.25 is a constant and the material constants A, $\sigma_{R}$, Q, n are taken as the same value as in Equation (5).

## 3. Results and Discussion

### 3.1. Friction at the Tool/Workpiece Interface

**Figure 4:**
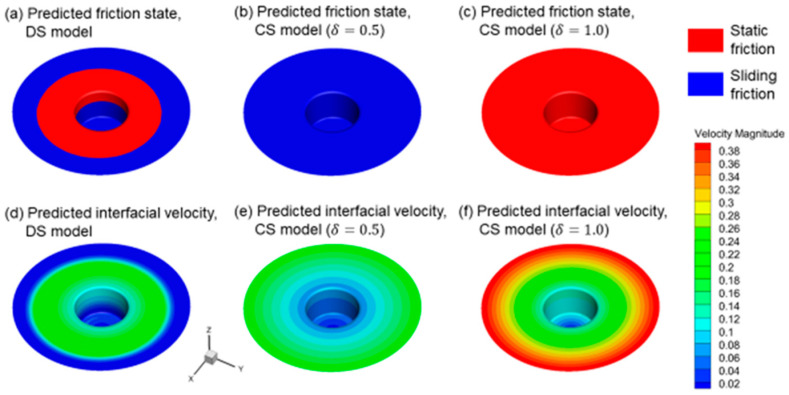
Effect of different interaction models on the predicted friction state and interfacial velocity. The present welding condition is 600 rpm, 40 mm/min, for FSW of 3 mm of AA2024.

An appropriate model for the interfacial friction between the welding tool and the workpiece is important for prediction of the combined thermal and mechanical condition and the material bonding behavior in the vicinity of the welding tool during FSW, as the interfacial friction is the underlying process that governs both the heat generation and the material flow. In Figure 4, the predicted friction state and the workpiece velocity at the interface using the DS model and the constant sticking (CS) model are plotted in Figure 4a–c and Figure 4d–f. The use of the DS model in the CFD simulation allows the automatic transition between the sticking state and slipping state at the interface, and thus the predicted contact state varies over the tool/workpiece interface, as shown in Figure 4a. What is noticeable is that a significant interfacial slipping occurs at the shoulder periphery and the bottom part of the pin, which results in slower flow velocity in the workpiece at these locations, as shown in Figure 4d. The capability of predicting a more realistic interfacial contact state is considered a major virtue of the DS model. In comparison, the contact state is considered as constant when the CS model is used, which is either slipping friction (Figure 4b) or static friction (Figure 4c), which may result in an over- or under-estimation of the flow velocity and deformation rate. In principle, the contact state at the tool/workpiece interface can be considered to be either sticking or slipping, based on the workpiece velocity. Generally, if the sticking presents at the interface, the workpiece velocity is considered to be very similar to that of the welding tool, which allows very fast material flow and minimum interfacial slipping; thus, much heat is generated from the high-rate plastic deformation. Otherwise, if slipping presents, less material flow will be caused, and in this case heat will be generated from the interfacial friction sliding.

### 3.2. Heat Generation and Temperature Field

**Figure 5:**
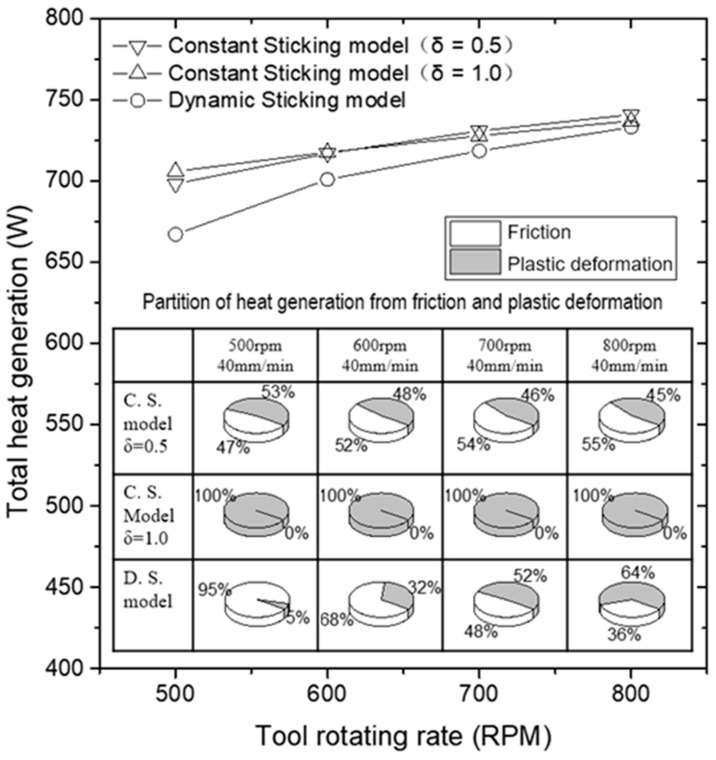
Predicted total amount and partition of the heat generation in FSW.

The heat generation in FSW is divided into the frictional heating and the plastic deformation heating. When integrating the heat flux calculated using Equations (9) and (10), the total heat generation is obtained. The total heat generation in different welding conditions is shown in Figure 5, together with the partition of heat generation from friction and plastic deformation. Obviously, the predicted heat partition changes significantly when the DS model is used. But, for the CS model, the partition of heat from the two types of heat flux at different welding conditions does not change much. Note that the total heat generation by the DS model is slightly less than that of the CS model, and the gap becomes greater when a slower too- rotating rate is used. The reason is that when more significant sliding friction presents at lower rotation rates, the plastic deformation heat is over-predicted by the CS model.

**Figure 6:**
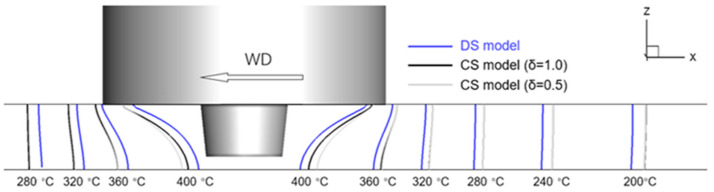
Temperature distribution at the longitudinal plane through the welding tool center (Y = 0). Tool rotation rate = 600 rpm, welding speed = 40 mm/min.

Figure 6 shows the predicted temperature distribution over a longitudinal plane (Y = 0) when the welding condition is 600 rpm and 40 mm/min. It could be found that the DS model and CS model result in very similar temperature profiles, while the CS models predict a slightly higher temperature. In order to further validate the temperature predictions, we compare the measured temperature history in Ref. [27] to our predictions with the same welding condition. Table 2 shows the peak temperatures and high-temperature hold times at different distances to the welding center using both experimental measurement and computational prediction. The comparison shows that our predictions of the temperature are valid and of acceptable precision in capturing the high-temperature hold time, which is very important for quantifying the temperature condition for the SSB. The CFD simulation with a CS model had been extensively validated in predicting the in-process temperature distribution [49,50]. Here, we show that the capability of the analysis of temperature filed persists when the new DS model is employed.

### 3.3. Material Flow Field in the Vicinity of the Welding Tool

**Figure 7:**
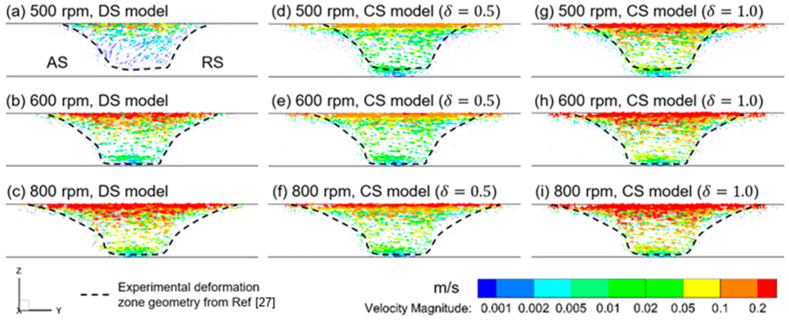
Vector plot of the predicted material flow field in the vicinity of the welding tool.

The original butting interface passes through the material flow zone in FSW, which provides the required thermal-mechanical condition for SSB. In order to predict the material flow behavior that changes with the welding parameter, the CFD simulation with the DS model is preferable to the prediction with the CS model. The predicted material flow fields of both the DS model and CS model at different welding parameters are shown in Figure 7a–i. It is notable that the region of significant plastic flow shrinks as the tool rotating rate decreases from 800 rpm to 500 rpm. This shrinkage in the flow region is caused by the increase in interfacial sliding at the tool/workpiece interface. In other words, sliding is present over a larger interfacial area, especially at the shoulder periphery and the bottom part of the pin, which causes significant reduction in material flow velocity in these locations. Thus, the flow region shrinkage occurs. In Figure 7, the predicted flow field is shown from the front view and is thus compared with the experimental measured deformation-zone profile with the corresponding welding condition [28]. It could be found that the DS model reveals the expected change in flow zone geometry, showing the material flow behaviors in the vicinity of the welding tool are highly dependent on the tool rotating rate. But this is not the case for the predicted flow field using the CS models. As mentioned above, the predicted heat generation and temperature using the CS model is shown to be higher than that of the DS model. It could be confirmed that material flow is over-predicted, especially at the shoulder periphery, where tool velocity reaches its maximum. 

### 3.4. Geometrical Evolution and Thermal-Mechanical History of the Butting Interface

**Figure 8:**
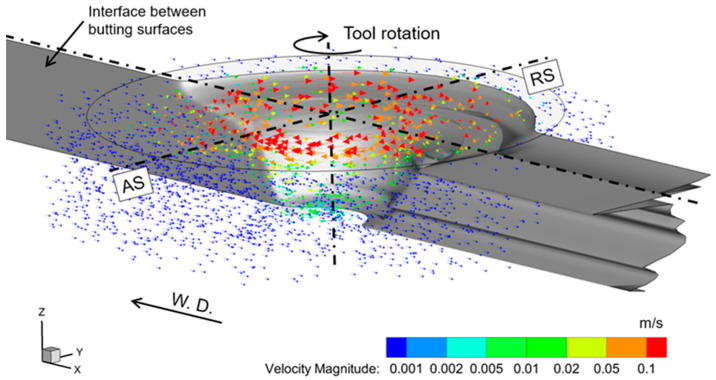
Geometric evolution of the original interface between the butting surfaces when passing through the material flow field (600 rpm, 40 mm/min). AS denotes the advancing sides. RS denotes the retreating side. W.D. denotes the welding direction.

Based on the simulated material flow field using the CFD simulation, the geometric evolution of the butting interface is analyzed by means of the proposed BIT method. Figure 8 plots the predicted geometric evolution of the interface between the butting surfaces when it passes the volume around the tool, where significant material flow occurs. Before FSW, the butting interface is assumed to be flat, and aligned with the tool center. It could be found that the butting interface starts to deform when it enters the flow zone in the vicinity of the welding tool and passes through the welding tool from the RS. The travel distances of the butting interface at different locations are different. For example, the material near the shoulder flows along paths with larger radii, compared to the material near the pin. The butting interface stops deformation in the rear side of the welding tool. It is worth noting that the points where the butting interface stops deformation are located at the edge of the high-velocity zone. As a result, after passing the flow zone, the butting interface is twisted, because of the variation in the flow paths at different depths. 

**Figure 9:**
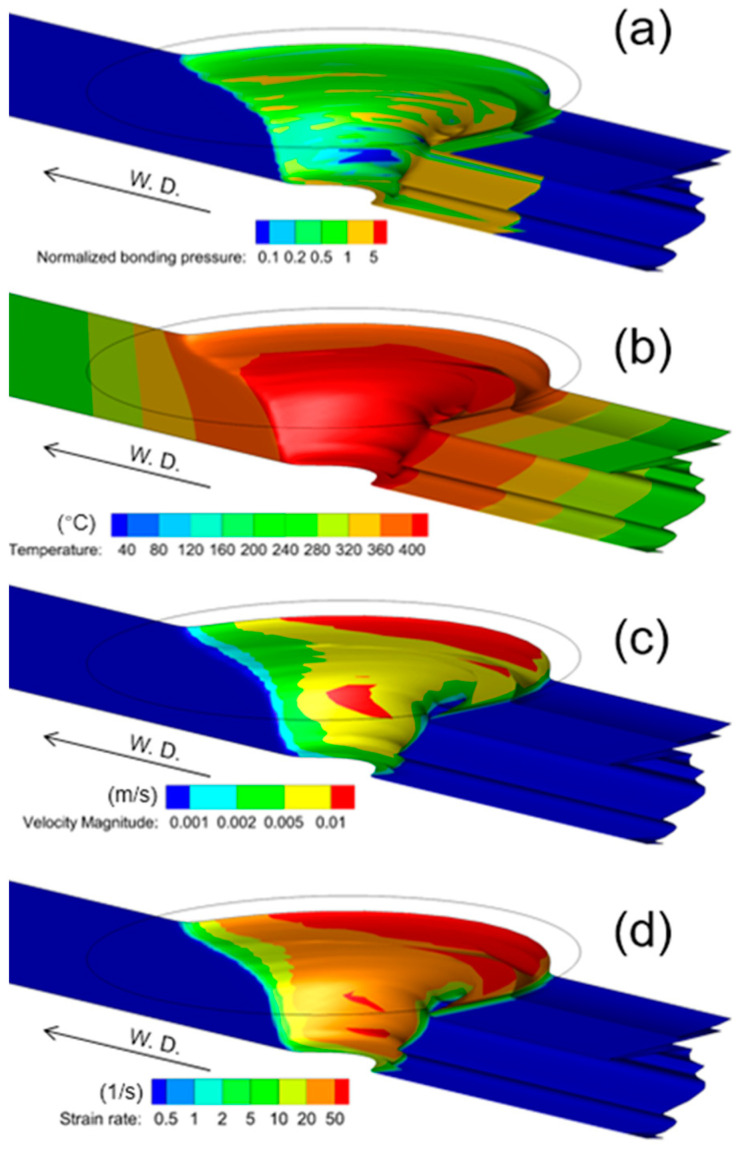
Thermal-mechanical history of the original interface between the butting surfaces when passing through the material flow field. (600 rpm, 40 mm/min). (**a**) normalized bonding pressure; (**b**) temperature; (**c**) velocity magnitude; (**d**) strain rate.

The thermal-mechanical state variables along the butting interface are responsible for the material bonding. Figure 9a–d show the evolution of the normalized bonding pressure, temperature, velocity magnitude and strain rate at the butting during FSW. The butting interface experiences a combined thermal and mechanical processing, including high pressure, elevated temperature, rapid plastic flow and severe plastic deformation, which is very complex. It could be found from Figure 9a that the normalized pressure at most of the butting interface ranges above 0.5, which means the bonding pressure at the butting interface reaches 0.5 times the local flow stress of the metal. Figure 9b shows that the temperature at the butting interface reaches generally above 360 °C when it passes through the welding tool vicinity. After the butting interface stops deformation, the temperature on it gradually decreases. Figure 9c depicts the distribution of flow velocity at the butting interface during FSW. The flow velocity on the butting interface generally reaches above 0.002 m/s. The velocity information, together with the flow path information, is used to evaluate the bonding time. Figure 9d plots the contour of the strain rate of the material at the butting interface, which is probed from the fluid zone. It could be found that the deforming butting interface is located inside the severe-deformation zone, and the strain rate reaches above 20/s. It is worth noting that, regarding the importance of these variables for analyzing the material bonding defects, the prediction accuracy might be checked and improved if a more advanced experimental observation method could be developed to validate this prediction. In order to develop an effective computational simulation for analyzing the material bonding in FSW, quantitative assessment of the predicted thermal-mechanical condition is required. 

### 3.5. Assessment of Bonding Quality Index for Analyzing Material Bonding in FSW

**Figure 10:**
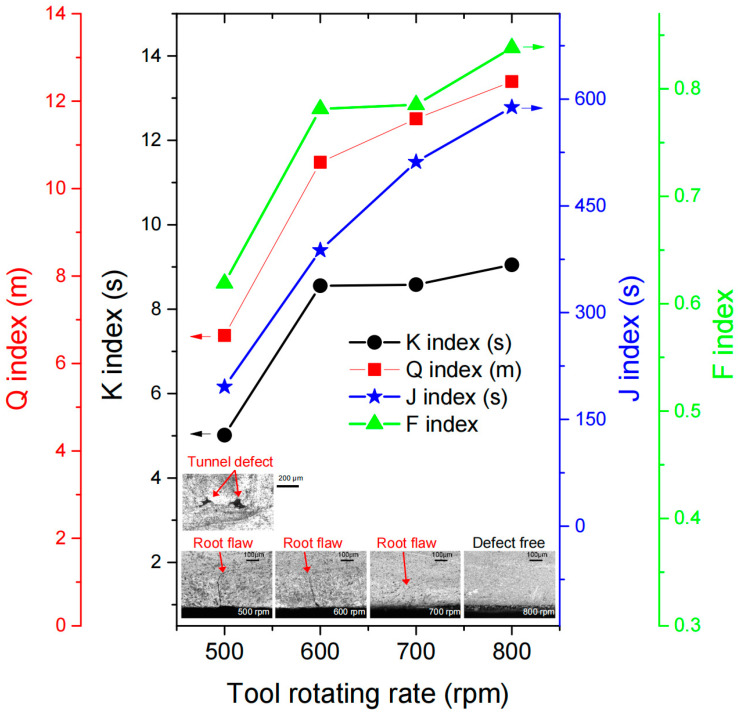
Bonding quality index (BQI) at weld root for different welding parameters. The insets show the microstructures at the weld root.

Assessment of the bonding quality by using a proper BQI is a key step in the development of an effective computational analysis for predicting the material bonding defects in FSW. The BQI generally measures how much the in-process thermal-mechanical condition is favorable for producing a sound SSB between the metal surfaces. Using the simulation methodology discussed above, the in-process thermal-mechanical conditions at the OBI with four different welding conditions are analyzed. In this paper, the values of the BQI at the weld root, which is within 0.5 mm from the back surface, is calculated by using Equations (16)–(19). Figure 10 shows the BQI at weld root for different welding parameters. It could be found from Figure 10 that all the four BQIs increase with the tool rotating rate, which indicates a better quality of the friction stir weld regarding the bonding between the butting surfaces. The increase in tool rotating rate leads to an increase in heat input in the process [16]. There is currently no experimental measurement available for direct observation of the material bonding behaviors in the FSW process. Therefore, it is difficult to validate the predicted bond quality. However, it is possible to measure the length of root flaw, one typical type of material bonding defect. The microstructures shown in the inset of Figure 10 indicate that the root flaw was shortened as the tool rotation increases, and no root flaw is found when the tool rotation is at 800 rpm. All the four indexes could be good candidates for checking the bonding quality. The J-index and the Q-index are the best because of their resolution and sensitivity in evaluating the bonding condition for a large range of rotation rates. It could be learned from Figure 10 that, when the BQI is below a critical value, a bonding defect is generated. Follow this concept, the bonding defect can be predicted once a critical BQI is determined. For example, if the critical value of the J-index is taken as 400, the bonding defects in the real welds can be predicted. 

By using the methodology in the present study, the prediction of material bonding defects in FSW becomes highly feasible if the developed methodology is extended to quantitatively determine the critical value of the BQI, i.e., a bonding criteria, for SSB of different alloys. In future work, there is still much work to conduct to enable the accurate prediction of material bonding defects for more materials and more welding conditions, given the current limitations of the proposed methodology. These limitations stem from that fact that the required parameters, such as the critical value of the BQI, are generally unknown. In fact, the concept of analyzing the bonding quality using a quantitative index is also being applied in the studies of forming and extrusion. For example, many well-designed experiments [30,38] were devoted to studying the critical thermal-condition for material bonding by physical simulation of the extrusion process. Similar studies can also be carried out to determine the critical bonding condition for FSW, to determine the critical BQI predicting the material bonding defect. 

## 4. Summary and Conclusions

This paper presents a computational methodology for predicting the material bonding defects during FSW of AA2024 using quantitative prediction and evaluation of the in-process thermo-mechanical condition. The following conclusions are obtained. 

(1) The developed analysis has three steps, which are (i) the thermal-mechanical simulation of FSW, (ii) the probing of the responsible thermal-mechanical state variables for material bonding, and (iii) the assessment of the responsible thermal-mechanical condition for material bonding. As an example, the methodology is successfully applied in the FSW of AA2024.

(2) The CFD model is used to evaluate the three-dimensional thermal-mechanical condition. The DS model at the tool/workpiece interface is shown to compare favorably to the DS model, regarding the accuracy in predicting the flow field. 

(3) A butting interface tracking (BIT) method is proposed. This method allows the analysis of the morphology of the initial butting interface during the welding process, which determines the specific temperature and bonding pressure at the butting interface for analysis of bonding quality.

(4) The performance of four bonding quality indexes (BQI) in predicting the material bonding defects are compared. Compared to other BQIs, including the Q-index and K-index, the J-index and F-index are more sensitive to the change in welding parameter. 

(5) The feasibility of the methodology is validated by the successful prediction of the root flaw defect in friction stir welds of AA2024. 

## Figures and Tables

**Table 1:** Temperature-dependent coefficient of friction (COF) [28].

Temperature (°C)	Coefficient of Friction
25	0.6
397	0.6
427	0.5
437	0.25
502	0.25

**Table 2:** Comparison between the measured and predicted characteristics of temperature history. The measured temperature history is taken from the authors’ previous work [27].

Distance to the Welding Center	Quantities	Measured Value	Predicted Value
3 mm	Peak temperature	419.4 °C	442.3 °C
	$t_{300{}^{\circ}C}$ *	55.9 s	59.8 s
6 mm	Peak temperature	396.1 °C	398.7 °C
	$t_{300{}^{\circ}C}$	48.9 s	51.8 s
9 mm	Peak temperature	347.5 °C	328.5 °C
	$t_{300{}^{\circ}C}$	44.8 s	32.7 s

* $t_{300{}^{\circ}C}$ represents the hold time when the temperature is above 300 °C.

## Data Availability

The data are available on request.

## Abbreviations

FSW	friction stir welding.
SSB	solid-state bonding.
CAE	computer-aided engineering.
BIT	butting interface tracking
CFD	computational fluid dynamics
CS	constant sticking
DS	dynamic sticking
CSRR	contact shoulder radius ratio
BQI	bonding quality index
UDF	user-defined functions
CoF	coefficient of friction
